# An investigation of cancer survival inequalities associated with individual-level socio-economic status, area-level deprivation, and contextual effects, in a cancer patient cohort in England and Wales

**DOI:** 10.1186/s12889-022-12525-1

**Published:** 2022-01-13

**Authors:** Fiona C. Ingleby, Laura M. Woods, Iain M. Atherton, Matthew Baker, Lucy Elliss-Brookes, Aurélien Belot

**Affiliations:** 1 Department of Non-Communicable Disease Epidemiology, Faculty of Epidemiology and Population Health, London School of Hygiene and Tropical Medicine, London, UK; 2 School of Health & Social Care, Edinburgh Napier University, Edinburgh, UK; 3 National Cancer Research Institute Consumer Forum, London, UK; 4 National Cancer Registration and Analysis Service, Public Health England, London, UK

**Keywords:** Cancer survival, Excess mortality hazard, Socio-economic status, Area-based deprivation, Contextual effect modification

## Abstract

**Background:**

People living in more deprived areas of high-income countries have lower cancer survival than those in less deprived areas. However, associations between individual-level socio-economic circumstances and cancer survival are relatively poorly understood. Moreover, few studies have addressed contextual effects, where associations between individual-level socio-economic status and cancer survival vary depending on area-based deprivation.

**Methods:**

Using 9276 individual-level observations from a longitudinal study in England and Wales, we examined the association with cancer survival of area-level deprivation and individual-level occupation, education, and income, for colorectal, prostate and breast cancer patients aged 20–99 at diagnosis. With flexible parametric excess hazard models, we estimated excess mortality across individual-level and area-level socio-economic variables and investigated contextual effects.

**Results:**

For colorectal cancers, we found evidence of an association between education and cancer survival in men with Excess Hazard Ratio (EHR) = 0.80, 95% Confidence Interval (CI) = 0.60;1.08 comparing “degree-level qualification and higher” to “no qualification” and EHR = 0.74 [0.56;0.97] comparing “apprenticeships and vocational qualification” to “no qualification”, adjusted on occupation and income; and between occupation and cancer survival for women with EHR = 0.77 [0.54;1.10] comparing “managerial/professional occupations” to “manual/technical,” and EHR = 0.81 [0.63;1.06] comparing “intermediate” to “manual/technical”, adjusted on education and income. For breast cancer in women, we found evidence of an association with income (EHR = 0.52 [0.29;0.95] for the highest income quintile compared to the lowest, adjusted on education and occupation), while for prostate cancer, all three individual-level socio-economic variables were associated to some extent with cancer survival. We found contextual effects of area-level deprivation on survival inequalities between occupation types for breast and prostate cancers, suggesting wider individual-level inequalities in more deprived areas compared to least deprived areas. Individual-level income inequalities for breast cancer were more evident than an area-level differential, suggesting that area-level deprivation might not be the most effective measure of inequality for this cancer. For colorectal cancer in both sexes, we found evidence suggesting area- and individual-level inequalities, but no evidence of contextual effects.

**Conclusions:**

Findings highlight that both individual and contextual effects contribute to inequalities in cancer outcomes. These insights provide potential avenues for more effective policy and practice.

**Supplementary Information:**

The online version contains supplementary material available at 10.1186/s12889-022-12525-1.

## Background

Research has shown that people living in less deprived areas of high-income countries experience lower mortality and longer life expectancy than those in more deprived areas [1, 2], and such inequalities are also found for cause-specific health outcomes for various diseases, including many types of cancer [3–7]. In the UK, the NHS (National Health Service) has highlighted the importance of reducing socio-economic health inequalities in its recent long-term plan [8], which dedicates funding and resources to narrowing inequalities over the next decade.

The majority of research on these inequalities has focused on differentials measured at an aggregated geographical level (largely due to aggregated data being more accessible than individual data), but some studies have also found that inequalities in mortality exist across individual-level socio-economic groups. For example, it has been shown that individuals on higher incomes or with a higher level of qualifications have lower mortality and longer life expectancy [9–11]. In order to successfully target the underlying causes of health inequalities, there is a need to fully understand to what extent differentials are due to area-level factors such as resource distribution, and to what extent they are associated with individual-level factors such as occupational health or personal circumstances affecting ability to access healthcare.

Moreover, there is also a need to explore the potential for area-level and individual-level factors to interact with one another, such that individual-level health inequalities could differ depending on the deprivation context of the area an individual lives in. In this way, data that describes area-level, or group-level, characteristics such as overall level of deprivation or access to services could have differing effects on individual outcomes according to personal characteristics such as education level or type of occupation. These ‘contextual effects’ could be particularly important given recent evidence that the area-level deprivation where an individual lives is not necessarily a good indicator of their individual socio-economic circumstances [12, 13]. An over-reliance on research that focuses only on area-level patterns of inequalities risks overlooking subsets of individuals who, for example, live in a relatively affluent area but have a low personal income and experience health inequalities differently from low income individuals who live in low income areas. There is also a risk that area-level differentials are interpreted principally at an individual level and the influence of the area itself is not acknowledged.

Contextual effects on all-cause mortality and overall health have been examined in several countries via a combined analysis of area-level and individual-level socio-economic measures [14–16], as well as some studies of contextual effects on cancer-specific mortality [17–20]. However, the effect of deprivation context on cancer survival in the UK remains poorly understood. This is the case despite the potential for such research to inform more effective health policy, especially in the face of evidence that there has been little, if any, improvement in socio-economic inequalities in cancer outcomes in recent years [4, 7, 21].

Using population-based survival data for patients diagnosed with colorectal, prostate, and breast cancer patients, we aimed (1) to quantify the association between individual-level socio-economic variables, area-based deprivation, and cancer survival, and (2) to investigate whether individual socio-economic survival differentials vary depending on the area-level deprivation context. We discuss the results in terms of gaining a better understanding of the underlying mechanisms of these socio-economic inequalities and the implications for health policy.

## Methods

### Data

We analysed data from the Office for National Statistics Longitudinal Study (ONS-LS), a long-term census-based multi-cohort study. The ONS-LS uses four annual birthdates as random selection criteria, giving a 1% sample of the England and Wales population [22]. Census data for cohort members are available from the 1971 census to the 2011 census. In addition, the data can be individually linked to external data, including cancer registrations and deaths registrations, as used in this analysis. Our analysis cohort included ONS-LS members who were present at either or both of the 2001 and 2011 census, and who had a first primary malignant cancer diagnosis registered between 1 January 2008 and 20 April 2016 for one of three major cancer sites: breast (ICD-10 code C50), prostate (C61), or colorectal (C18–21). These cancer sites were selected because area-level deprivation differentials in cancer survival (as opposed to differentials in cancer incidence) have previously been identified [4], as well as having sufficiently large case numbers to enable the analysis models described here. Individuals aged between 20 and 99 at the time of diagnosis were included. For each patient, we calculated the time from diagnosis to either date of death or date of censoring on 31 December 2017 (the date of the most recent linkage to the deaths registry data).

#### Individual-level socio-economic variables

At an individual-level, data from the 2011 census was used to group individuals according to three separate socio-economic variables: income, education and occupation. These three variables are commonly used in the social sciences to summarise the wide range of socio-economic circumstances an individual can experience [23].

Education was categorised as one of four groups according to the standard levels of England and Wales qualifications used in the census: (1) no qualifications; (2) school-level qualifications such as GCSEs and A-levels; (3) apprenticeships and vocational qualifications; and (4) degree-level education and higher. The wording of the census includes equivalent types of qualification held by individuals not educated in the English or Welsh system.

Occupation was categorised as one of three broad types of occupations, using three-group version of the National Statistics Socio-Economic Classification (NS-SEC): (1) technical, routine and manual occupations; (2) intermediate occupations; and (3) higher managerial, administrative and professional occupations [24].

Individual income was estimated indirectly from census data on an individual’s age, sex, and Standard Occupational Classification (SOC) code, using an externally-validated linear model prediction method described by Clemens and Dibben [25]. In addition, we carried out a data-driven adjustment of income for individuals aged over 60 who were most likely to be retired. This adjustment used observed annualised percentage decreases in income for over-60 year olds, as reported in the English Longitudinal Study of Ageing [26]. Income estimates were grouped into quintiles separately for each sex. Quintiles were calculated on the full ONS-LS cohort, prior to selection of only cancer patients for the analysis. Income estimates were therefore linked to occupation, however, the use of SOC codes rather than NS-SEC (as for the occupation variable above) means that these variables are independent of one another, since SOC codes are linked to specific jobs, as opposed to the broad NS-SEC categories for types of occupation.

#### Area-level deprivation variable

As a measure of area-based deprivation, we used the income domain of the Index of Multiple Deprivation (IMD) as calculated for each Lower-level Super Output Area (LSOA) in England and Wales. These geographical units are commonly used in UK studies based on routinely collected administrative data and represent lower-level geographical units with a typical population of around 1000–1500 people. The income sub-index is calculated using data on the number of residents per LSOA recorded as not earning or on low incomes resulting in qualification for in-work benefits. The LSOA of residence for each ONS-LS member was recorded directly in the 2011 census, and could be derived indirectly for the 2001 census, using district code and ward of residence. For each individual, area-based deprivation was linked from the census date temporally closest to the cancer diagnosis. We used the published area-level deprivation data temporally closest to each census: for the 2001 census this was the English IMD 2004 [27] and the Welsh 2005 report [28], and for the 2011 census this was the English IMD 2015 [29] and the Welsh 2014 report [30]. LSOA deprivation score was linked to each individual as a ventile (i.e., a similar concept to a quintile, but with 20 equal quantiles instead of 5, to provide more granular analysis). Ventiles were ranked by numbering each from 1 (most deprived) to 20 (least deprived). This scale was used instead of raw deprivation scores in order to avoid potential individual identifiability while capturing as much information about the area-level deprivation gradient as possible from the continuous score. This ventile rank was analysed as a continuous variable in the statistical models and for the purposes of conciseness is referred to throughout as the ‘area-level deprivation’ variable.

### Analyses

Out of a total of 10,009 cancer patients in the ONS-LS fulfilling our patient cohort criteria, socio-economic data were unavailable for 7%, who were excluded from the analysis. The remaining 9276 individuals were included in the analysis. From this analysis cohort, data from the 2011 census were fully available for 91% with a further 4% of the cohort classifiable on the basis of the 2001 census (the majority of these individuals had a cancer diagnosis between 2008 and 2011 and died prior to the 2011 census date). The remaining 5% of individuals had data for at least one socio-economic variable completed by proxy using another adult resident (where data was available) in the same household (usually household head). The total numbers of patients and numbers of deaths included in the analyses are shown in Table 1.

**Table 1:** Patients by cancer site, age group, and individual-level socio-economic group; and deaths at 1-year and 5-years after diagnosis. Data source: ONS-LS

Category		Men Colorectal		Women Colorectal		Men Prostate		Women Breast	
		N	%	N	%	N	%	N	%
**Age group**	20–54	142	9%	130	10%	109	4%	1050	31%
	55–64	325	22%	233	19%	624	20%	812	23%
	65–74	486	32%	343	28%	1240	41%	834	24%
	75–99	569	37%	531	43%	1071	35%	777	22%
**Education**	No qualifications	611	40%	598	48%	1113	36%	1197	34%
	School-level	335	22%	333	27%	641	21%	1191	34%
	Apprent/Vocat	280	18%	76	6%	503	17%	241	7%
	Degree-level	296	20%	230	19%	787	26%	844	25%
**Occupation**	Manual/Tech	671	44%	560	45%	1179	39%	1320	38%
	Intermediate	353	23%	348	28%	712	23%	1087	31%
	Manag/Prof	498	33%	329	27%	1153	38%	1066	31%
**Income**	Lowest income	402	26%	339	27%	737	24%	615	18%
	Q2	375	25%	355	29%	692	23%	854	25%
	Q3	357	23%	223	18%	725	24%	706	20%
	Q4	223	15%	200	16%	511	17%	720	20%
	Highest income	165	11%	120	10%	379	12%	578	17%
**Deaths**	1-year	350	23%	297	24%	213	7%	174	5%
	5-year	715	47%	569	46%	700	23%	625	18%
	**Total**	**1522**		**1237**		**3044**		**3473**	

We used a relative survival approach, in which the overall mortality hazard is expressed as the sum of a population (expected) mortality hazard and an excess mortality hazard (EMH). The EMH is the quantity of interest and is interpreted as the mortality hazard due directly or indirectly to the relevant cancer site [31–33], while the expected mortality hazard is considered known and is obtained from appropriate life tables derived from the population from which the patients are drawn. We aimed to model the EMH as a flexible function of time since cancer diagnosis as well as with respect to the variables of interest. All statistical models were carried out separately for men and women and for each cancer site. The ‘mexhaz’ analysis package for excess hazards survival modelling [34–36] was used for all analyses with R statistical software v.3.6.3. This analysis approach allows for multi-level models where needed to account for clustering by geographical area. However, an initial review of the data showed that a single-level analysis approach was sufficient here, as almost all individuals were unique to their LSOA within each cancer site. This applied to 96% of the analysis cohort; with the remaining 4% at a maximum of two individuals in the same geographic area. We therefore adopted a single-level modelling approach in this study.

#### Expected mortality rates

We obtained population-specific expected background mortality rates from a Poisson regression model (with natural cubic splines) of overall mortality, using the same ONS-LS 2011 census cohort (not restricted to cancer patients), and following the methods as described in [11, 37]. From this model, we linked individuals to the expected mortality estimate specific to their age at date of death or censoring, calendar year at date of death or censoring, sex, and individual socio-economic variables.

#### Modelling approach

The first analysis aim was to quantify the association between individual-level socio-economic variables and EMH (or equivalently, cancer survival). To do this, EMH was modelled against age at diagnosis and the three categorical individual-level socio-economic variables (education, occupation and income), assuming proportional hazards for the socio-economic variables. The baseline EMH was parametrised with a B-spline of degree 3 and a knot located at 1 year. Since age is an important prognostic factor of EMH [38], we investigated four alternative models with different regression assumptions for the functional form of age: (i) a simple linear effect; (ii) with a non-linear effect; (iii) with a linear but time-dependent effect; and (iv) with non-linear and time-dependent effects. We identified the model with the lowest Akaike Information Criteria (AIC) as that with the best fit (Supplementary Materials, Table S1). In order to quantify the evidence for the association between each individual-level socio-economic variable (education, income, and occupation) and the EMH, we sequentially removed each individual-level socio-economic variable from this best fit model, and used likelihood ratio tests to compare models with and without each socio-economic variable. We present the likelihood ratio test statistic and associated *p*-value as supplementary measures.

The second aim was to investigate whether individual-level socio-economic survival differentials varied across area-level deprivation contexts. For this, the best model for each sex/cancer site combination as identified above from the individual-level analysis for Aim 1 was taken forward to a second stage of modelling, where area-level deprivation was added as a continuous variable (assuming linear functional form), as well as interaction terms. For each dataset, we fitted eight alternative models (Supplementary Materials, Table S1), one for each possible combination of interactions between the area-level deprivation variable and the individual-level socio-economic variables. These interactions represent the contextual effect of area-level deprivation on cancer survival across the individual-level socio-economic groups. The model with the lowest AIC was identified as the best fit for the data and used for interpretation of the results.

Estimates of net survival and excess hazard were predicted from the EMH regression model coefficients. Where estimates are shown within the most and least deprived area contexts, the first and twentieth area-level deprivation ventiles, respectively, are used. Net survival (ie, cancer survival in our study) represents the survival probability of cancer patients after accounting for the other causes of death, and is used to quantify inequalities on an absolute scale. We also report excess hazard ratios (EHR) to allow comparisons across groups on a relative scale, using the individual-level most socio-economically deprived groups as the baseline. Excess hazard ratios for the area-level deprivation represent the increment for a one ventile increase in the area-level variable.

### Ethics

Ethics approval for this study was granted by the London School of Hygiene and Tropical Medicine Ethics Committee via online application (approved 01/02/2018).

## Results

The analysis cohort included 1522 men and 1237 women with colorectal cancer, 3044 men with prostate cancer, and 3473 women with breast cancer. The distributions of patients across socio-economic groups, and numbers of deaths, are shown in Table 1. The cohort was broadly representative of the age distribution of cancer patients in the whole population of England and Wales (Supplementary Materials, Table S2). A preliminary model fitted with only age and the area-based deprivation variable showed that those living in less deprived areas experienced lower excess hazard than those in more deprived areas for prostate (EHR = 0.80 [95% confidence interval, CI:0.72–0.90]), breast (EHR = 0.89 [0.82–0.96]), and colorectal cancers (men: EHR = 0.95 [0.89–1.02]; women: EHR = 0.95 [0.89–1.03]).

### Aim 1: individual-level effects

**Fig. 1:**
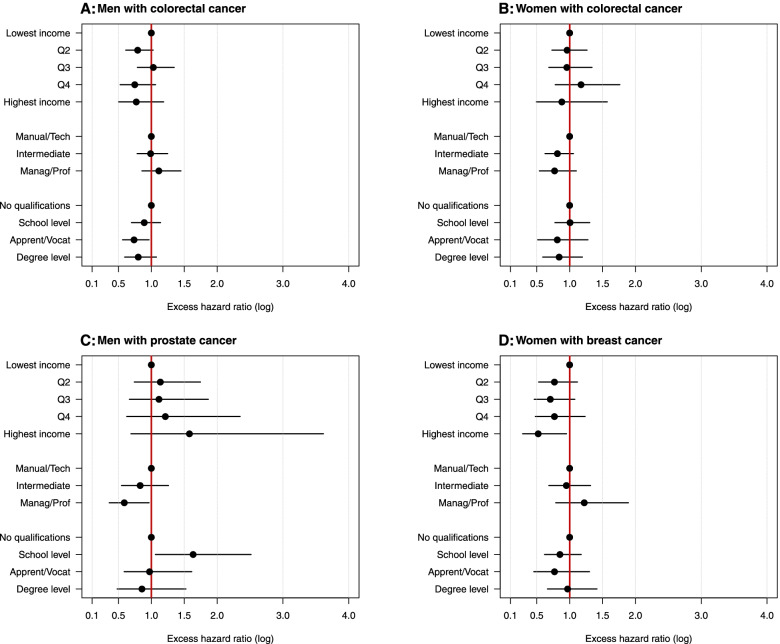
Modelled excess hazard ratios (95% CI) by sex and cancer site across individual-level socio-economic groups as obtained from the model with best fit for Aim 1 (see methods). Data source: ONS-LS

Trends across individual-level socio-economic groups were mixed, as shown by the excess hazard ratios (unadjusted for any area-level effects but adjusted for other individual effects) in Fig. 1 for each sex/cancer site combination (also shown in Table S3). There was some evidence that individuals in managerial/professional occupations experienced lower excess mortality than other occupation types for prostate cancer (likelihood ratio test, LRT = 4.64; *P* = 0.098; Table S4) and for colorectal cancer in women (LRT = 4.70; *P* = 0.095), after adjusting for age and the other individual-level socio-economic variables. Additionally, there was some mixed indication that individuals with degree-level education or apprenticeships tended to experience lower EMH than those with no qualifications or school-level qualifications for colorectal cancer (LRT = 6.25; *P* = 0.100) and prostate cancer (LRT = 8.93; *P* = 0.030) in men. For women with breast cancer, individuals in higher income quintiles tended to have lower EMH than those on lower incomes, after adjusting for age, education and occupation (LRT = 8.89; *P* = 0.064). The opposite was the case for men with prostate cancer: men in higher income quintiles tended to have higher EMH than those on lower incomes after adjusting for age, education, and occupation (LRT = 7.79; *P* = 0.099).

### Aim 2: contextual effects

**Fig. 2:**
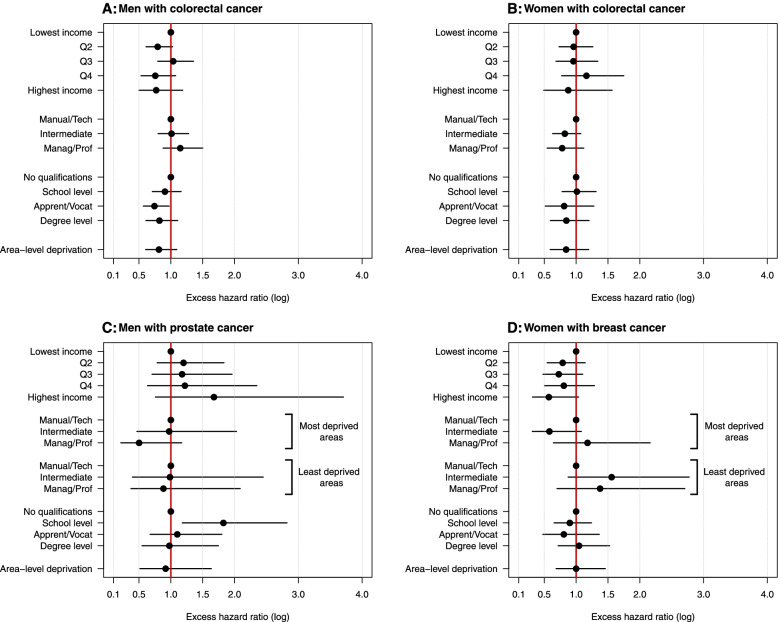
Modelled excess hazard ratios (95% CI) by sex and cancer site across individual- and area-level socio-economic groups as obtained from the model with best fit for Aim 2 (see methods). Note: excess hazard ratios across occupation groups for prostate and breast cancers are shown within the least and most deprived deprivation ventiles. Data source: ONS-LS

**Fig. 3:**
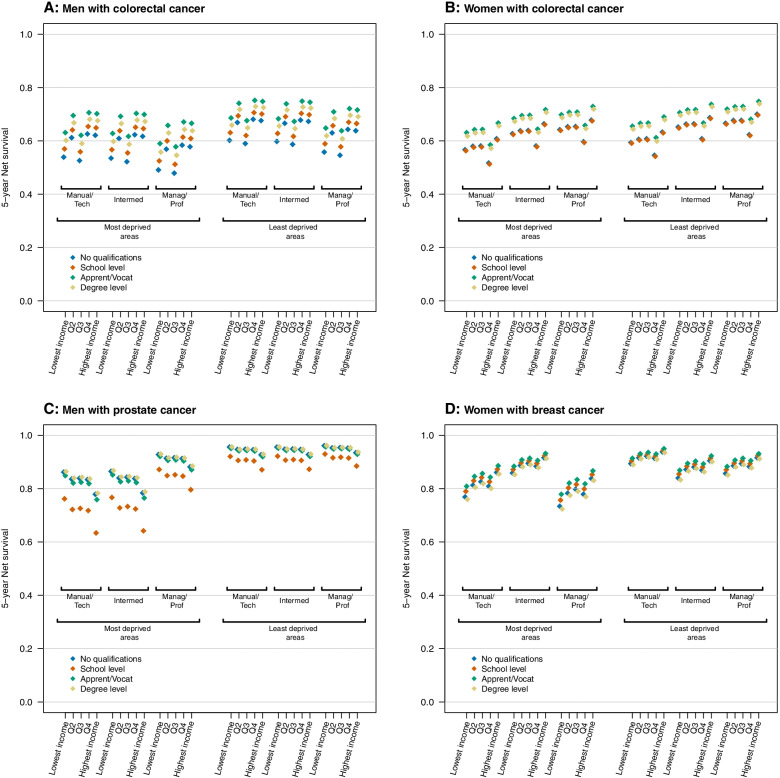
Five-year net survival estimates for individual-level socio-economic groups according to area-level deprivation, as obtained from the model with best fit for Aim 2 (see methods), and for patients aged 70 years at diagnosis. Data source: ONS-LS

For men with colorectal cancer, there was no evidence of contextual effect modification of individual-level socio-economic inequalities across area-level contexts. Excess hazard ratios across the individual-level socio-economic groups after adjustment for area-level effects are shown in Fig. 2A (also shown in Table S5). The lowest 5-year net survival was observed for those with no qualifications, in managerial/professional occupations, and in the middle income quintile, and this was true both in the most deprived (NS = 48% [36–59%]) and least deprived areas (NS = 55% [44–64%]; Fig. 3A and Table S6). The highest net survival was observed for those with apprenticeship/vocational qualifications, manual/technical occupations, and in the 4th highest income quintile (Fig. 3A). Again, this was the same in the most deprived (NS = 71% [60–79%]) and least deprived areas (NS = 75% [65–83%]).

Similarly, no contextual effect was observed for women with colorectal cancer (Fig. 2B). The highest net survival observed in each area-level context was for individuals with apprenticeship/vocational qualifications, managerial/professional occupations, and in the highest income quintile (NS = 73% [55–85%] for the most deprived areas; NS = 75% [58–86%] for the least deprived areas; Fig. 3B). The lowest net survival was for those with school-level qualifications, manual/technical occupations, and in the 4th highest income quintile for both the most deprived areas (NS = 51% [36–65%]) and the least deprived areas (NS = 54% [38–68%]; Fig. 3B).

For men with prostate cancer, there was evidence that area-level context modified individual-level occupational differences. Men in managerial/professional occupations experienced lower excess hazard than men in other occupational groups, and this inequality was wider in the most deprived areas (EHR = 0.50 [0.22–1.17]) compared to the least deprived areas (EHR = 0.88 [0.37–2.09]; Fig. 2C). Net survival estimates showed that the absolute difference between groups with lowest and highest survival in the most deprived areas was 29.6%, compared to 9.1% in the least deprived areas (Fig. 3C).

There was also evidence of a contextual effect of area-level deprivation on occupation for women with breast cancer. Women with intermediate occupations had a relatively high excess hazard in the least deprived areas (EHR = 1.56 [0.87–2.77]) compared to a relatively low excess hazard in the most deprived areas (EHR = 0.58 [0.31–1.08]; Fig. 2D). Survival differentials between individual-level groups in Fig. 3D were wider in the most deprived areas (20.8%) than in the least deprived areas (11.7%). This difference was less pronounced than that observed for prostate cancer.

The adjusted models including both individual-level and area-level effects showed that the evidence for a lower excess hazard in less deprived areas was weakened for both prostate cancer (EHR = 0.92 [0.52–1.64]) and breast cancer (EHR = 0.98 [0.69–1.46]) after adjusting for individual socio-economic variables, as compared to the unadjusted area-level effects reported above. The opposite was true for colorectal cancer: area-level deprivation inequalities were wider once adjusted for individual-level effects (men: EHR = 0.81 [95% CI: 0.60–1.09]; women: EHR = 0.84 [0.59–1.19]).

## Discussion

Our analyses have identified mixed evidence of individual-level socio-economic inequalities in cancer survival, as well as evidence of contextual effect modification for breast and prostate cancers but not for colorectal cancer. Adjusting for area-based deprivation hardly impacted the association between individual socio-economic variables and the EMH for colorectal cancer. Conversely, for prostate and breast cancers, the contextual effects we found widened individual-level inequalities between occupation groups depending on the level of area-based deprivation. We also found that, for prostate and breast cancers, the disadvantageous effect of area-based deprivation was substantially reduced by adjustment for individual-level effects, whereas the opposite was true for colorectal cancer.

We used a relative survival approach for the analysis, which enabled the association between socio-economic circumstances and excess (cancer-specific) mortality to be assessed independently of expected (background) mortality [31–33]. The estimates of background mortality used here were based on the same population cohort (but not restricted to cancer patients), and we have previously shown that, within this cohort, there are wide inequalities in all-cause mortality and adult life expectancy between individual-level socio-economic groups [11]. Here, by accounting for these differences in background mortality using a relative survival approach, we have focussed our analyses specifically on the association between individual socio-economic status and EMH. Our analysis does not however account for important prognostic factors such as stage of cancer at diagnosis, or treatment undertaken, which are not routinely collected in the ONS-LS dataset, and so these factors could contribute unmeasured confounding effects to our results.

We note that the net survival and EHR estimates from our models have wide confidence intervals for some sex/cancer combinations, as presented in the results. This is likely due to use of a relatively small sample size combined with relatively complex models. Our model selection approach towards examining evidence for contextual effects gives good support for the overall presence and absence of the contextual effects described here, but the wide Wald-type confidence intervals around the EHR estimates for specific socio-economic sub-groups means that we can only describe the observed trends contributing to the contextual effects. On a related note, we would like to point out that we were unable to find evidence of an association between some individual-level socio-economic variables and excess mortality hazard, but it does not mean that such associations do not exist. We also note that all estimates are based on the model with the lowest AIC. The models with an AIC within 2 units of the lowest AIC (Table S1) could also be supported by the data, and so multi-model inference would be an interesting research avenue for further analysis [39].

The differentials observed here varied across the different individual-level socio-economic variables included in the analysis. Disparities were most notable according to occupation type for men with prostate cancer and women with colorectal cancer; across income quintiles for women with breast cancer; and across education groups for men with colorectal and prostate cancers. In addition, there was a slight trend for higher EMH in prostate cancer patients associated with higher income. Although unexpected, this trend could be partially explained by the analysis adjusting for individual-level occupation and education, which are likely to be linked to income. Generally, it might be expected to observe individual-level socio-economic inequalities that consistently reflect the well-documented area-level deprivation differentials [3–7]. However, the individual-level variables were based on specific dimensions of socio-economic status, as opposed to the summary deprivation scores used at an area-level, and as such the observed mixed results might suggest that the specific underlying mechanisms of individual-level inequalities can differ between cancer sites.

We found evidence of persistent area-level deprivation inequalities for colorectal cancers in the models that included both individual socio-economic variables and area-level deprivation. Previous literature has comprehensively shown that cancer survival is lower in more deprived areas [3–7], but the increase of area-based deprivation inequalities even after adjustment for individual patient characteristics in this analysis is novel. Conversely, the lack of area-level deprivation inequalities for breast and prostate cancers after adjusting for individual-level effects was unexpected, given area-level differentials documented previously for breast [4] and prostate cancer [40], and could suggest that these inequalities are more effectively explained by individual-level variables. Although the variables analysed here might not necessarily explain the inequalities directly, they might act as a more appropriate proxy for the underlying mechanism. For example, inequalities in breast cancer survival could be partially explained by individual characteristics (such as BMI) influencing treatment pathways, and so focussing on individual-level characteristics to estimate inequalities might be more appropriate than the use of area-level scores in certain cases.

The models used here enabled us to simultaneously investigate individual socio-economic status and contextual effects for cancer survival, while properly adjusting for age. To our knowledge this has not been explicitly examined in a UK population setting to date. For prostate cancer, and to some extent for breast cancer, there was evidence that inequalities in cancer survival across individual-level occupation groups were wider in more deprived areas than in less deprived areas. Although some of this effect could be accounted for by a ceiling effect, in which differences between groups will appear increasingly smaller as survival approaches 100%, it is unlikely this phenomenon explains all of the wide differences observed here, particularly for prostate cancer. The observed contextual effect amplifies inequalities, such that the sub-group of individuals in manual/technical occupations who live in the most deprived areas experience a survival disadvantage in addition to that previously estimated in area-level analysis. This differential is not detectable in studies that use only area-based socio-economic metrics. As a result, policies relying exclusively on such studies may overlook these individuals.

In addition to widening inequalities in the most deprived areas, the contextual effect observed for women with breast cancer indicated differences in terms of which occupational groups experienced the highest cancer survival in different area-level deprivation contexts. It is unclear from this analysis why this might occur. It is possible that unmeasured confounding could help to explain this effect, for example, if women in manual/technical occupations living in less deprived areas tend to be in households that are generally less deprived due to the income or occupation of their partner. Further research could consider the underlying reasons for these patterns. Analysis of household income as opposed to individual income may be useful to consider. However in this study, data pertaining to non-ONS-LS members of the relevant households is not widely available. This precluded the estimation of household total income.

For colorectal cancer for both men and women, there was no evidence of any contextual effect modification due to area-level deprivation, whilst there was stronger evidence of area-level inequalities. There was also some indication of individual-level inequalities across education and income groups for men, and across occupation groups for women. These results suggest that individual- and area-level deprivation exert independent effects on cancer survival, and future healthcare policy is likely to benefit from accounting for both these potential sources of inequality.

A major reason for the relative lack of research on individual-level and contextual effects on health outcomes, especially in a UK setting, is that individual-level data is protected and aggregated area-level data are far more accessible. Studies such as the ONS-LS offer rich individual-level data that could be used for follow-up work in this area, and the ONS-LS is particularly useful in this respect due to its representativeness of the overall population [22]. In spite of this, the cohort is based on only approximately 1% of the whole population, and so when focussed to individual cancer sites, sample sizes are not large enough to consider less common cancers, nor large enough to incorporate more complex effects such as modelling non-proportional hazards for the socio-economic variables. Further work should consider ways to adapt these methods to extend the generalisability and detail of the results. Although it is possible to group cancer sites for analysis, this would need to be done with caution, since we have shown here that contextual effects can differ between cancer sites. These differences are likely to be biologically meaningful, given the disease-specific differences in screening, diagnosis, and treatment, meaning that combining cancer sites for analysis is unlikely to be appropriate.

Contextual effects on cancer outcomes have been explored to some extent in other countries [15–20, 41]. Research in the USA has found evidence of context-specific inequalities and support for multi-level health policy interventions relating to breast, colorectal and prostate cancers [18–20]. These are consistent with our results for prostate and breast cancers, although we find no evidence for contextual effects for colorectal cancer in this population cohort. A study focused on prostate cancer outcomes in a Californian population found individual-level inequalities across education groups consistent with the survival disadvantage we identified here for men with school-level education, and in addition they found contextual effects of these inequalities [20]. Although our study found evidence of contextual effects across occupational types rather than educational level for men with prostate cancer, education level and occupation type are correlated so further research could explore the underlying causes of these contextual effects in more detail. Furthermore, a systematic review based on USA data highlighted the focus on common cancer sites for such analyses, reinforcing the point that there is a need for studies to be extended to consider contextual effects for less common cancers [19].

## Conclusions

In summary, this study has given novel insight into the complexities of socio-economic inequalities in cancer outcomes by identifying individual-level socio-economic inequalities alongside context-dependent effects for major cancer sites in a UK population setting. Our results lend support for health policy that considers both area-level and individual-level interventions, as well as taking context into account. For example, individuals with low education level may struggle more than others to navigate the NHS system smoothly and to understand the different treatment options due to poor health literacy. For occupation, job constraints such as working hours or inability to take paid sick leave could also influence and drive inequalities. These elements should not be forgotten when health policies are decided, and such policies should not be based on area-based measures of deprivation alone. That particular indicators of individual or contextual deprivation appear to impinge on very specific cancers points towards avenues of research which might lead to a fuller understanding of why certain groups experience poorer outcomes than others. Finally, our work also supports the view that practitioners should not rely only on postcode if looking for social determinants of health [42].

## Supplementary Information

**Additional file 1 **This document contains six supplementary tables, as referenced in the text. **Table S1** contains details of the alternative models examined during the model building stage of the analysis, and shows the model with the best fit that was used to interpret the main results. **Table S2** shows the age distribution of patients (by cancer site and sex) for the ONS-LS cohort used in the analysis, compared to the whole population of England and Wales. This information is used to assess representativeness of the study cohort. **Table S3** shows the excess hazard ratios estimated from the best models for analysis aim 1, and supports Fig. 1 in the main manuscript, which illustrates these same data. **Table S4** shows the results of the likelihood ratio tests of the individual socio-economic variables included in the models for analysis aim 1. **Table S5** shows the excess hazard ratios estimated from the best models for analysis aim 2, and supports Fig. 2 in the main manuscript, which illustrates these same data. **Table S6** gives the net survival (as estimated from the best fit model) for each cancer site and sex, across each of the individual-level socio-economic groups, within both the most and least deprived deprivation contexts. This data supports that shown in the main manuscript Fig. 3, and Table S6 additionally provides confidence intervals around each estimate.

## Data Availability

Data are not publicly available but can be accessed via appropriate application to the ONS Longitudinal Study. This work contains statistical data from ONS, which is Crown Copyright. The use of the ONS statistical data in this work does not imply the endorsement of the ONS in relation to the interpretation or analysis of the statistical data. This work uses research datasets which may not exactly reproduce National Statistics aggregates. All analysis code can be requested from the corresponding author.

## Abbreviations

CI: Confidence Interval
EHR: Excess Hazard Ratio
EMH: Excess Mortality Hazard
GCSE: General Certificate of Secondary Education
ICD: International Classification of Diseases
IMD: Index of Multiple Deprivation
LRT: Likelihood Ratio Test
LSOA: Lower-Level Super Output Area
NHS: National Health Service
NS: Net Survival
NS-SEC: National Statistics Socio-Economic Classification
ONS-LS: Office of National Statistics Longitudinal Study
SOC: Standard Occupational Classification
