# Supplementary material for: An investigation of cancer survival inequalities associated with individual-level socio-economic status, area-level deprivation, and contextual effects, in a cancer patient cohort in England and Wales
Source: BMC Public Health. 2022 Jan 13;22:90. doi: 10.1186/s12889-022-12525-1 (PMC8759193; doi:10.1186/s12889-022-12525-1)
Supplement: Supplementary file 1 — Additional file 1 This document contains six supplementary tables, as referenced in the text. Table S1 contains details of the alternative models examined during the model building stage of the analysis, and shows the model with the best fit that was used to interpret the main results. Table S2 shows the age distribution of patients (by cancer site and sex) for the ONS-LS cohort used in the analysis, compared to the whole population of England and Wales. This information is used to assess representativeness of the study cohort. Table S3 shows the excess hazard ratios estimated from the best models for analysis aim 1, and supports Fig. 1 in the main manuscript, which illustrates these same data. Table S4 shows the results of the likelihood ratio tests of the individual socio-economic variables included in the models for analysis aim 1. Table S5 shows the excess hazard ratios estimated from the best models for analysis aim 2, and supports Fig. 2 in the main manuscript, which illustrates these same data. Table S6 gives the net survival (as estimated from the best fit model) for each cancer site and sex, across each of the individual-level socio-economic groups, within both the most and least deprived deprivation contexts. This data supports that shown in the main manuscript Fig. 3, and Table S6 additionally provides confidence intervals around each estimate. [file 12889_2022_12525_MOESM1_ESM.docx]

**Supplementary Materials**

**Table S1.** Model AIC shown for alternative models compared in the model building process. Cells highlighted in grey show the best model fit. Data source: ONS-LS.

| **Model rationale** | **Dataset** | | | |
| --- | --- | --- | --- | --- |
|  | **Men**  **Colorectal** | **Women Colorectal** | **Men**  **Prostate** | **Women**  **Breast** |
| ***Aim 1*** | | | | |
| Linear effect of age | 3896.31 | 2920.02 | 5007.58 | 5314.64 |
| Non-linear effects of age | 3875.38 | 2914.64 | 5058.05 | 5371.3 |
| Linear & non-proportional effects of age | 3888.29 | 2884.03 | 5000.18 | 5299.44 |
| Non-linear & non-proportional effects of age | 3874.47 | 2887.76 | 5065.24 | 5355.39 |
|  |  |  |  |  |
| ***Aim 2*** | | | | |
| No contextual effect modification | 3875.00 | 2885.79 | 4993.82 | 5294.97 |
| Contextual effect modification on education | 3876.99 | 2889.22 | 4994.49 | 5299.47 |
| Contextual effect modification on occupation | 3877.92 | 2888.05 | 4993.06 | 5293.69 |
| Contextual effect modification on income | 3881.82 | 2887.81 | 4997.37 | 5300.49 |
| Contextual effect modification on education & occupation | 3879.78 | 2889.77 | 4997.96 | 5298.91 |
| Contextual effect modification on education & income | 3885.02 | 2888.58 | 5002.00 | 5304.93 |
| Contextual effect modification on occupation & income | 3884.79 | 2889.94 | 4999.92 | 5299.58 |
| Contextual effect modification on education, occupation & income | 3886.78 | 2890.49 | 5005.19 | 5304.09 |
|  |  |  |  |  |
|  |  |  |  |  |

**Table S2.** Distribution of cancer patients in the ONS-LS analysis cohort (N and %) compared to distribution (%) in the whole population of England & Wales, over the same analysis period, by age group, cancer site, and sex. Data source: ONS-LS and National Cancer Registry.

| **Age group** | **Men**  **Colorectal** | | | **Women**  **Colorectal** | | | **Men**  **Prostate** | | | **Women**  **Breast** | | |
| --- | --- | --- | --- | --- | --- | --- | --- | --- | --- | --- | --- | --- |
|  | **ONS LS**  **N** | **ONS LS**  **%** | **E+W**  **%** | **ONS LS**  **N** | **ONS LS**  **%** | **E+W**  **%** | **ONS LS**  **N** | **ONS LS**  **%** | **E+W**  **%** | **ONS LS**  **N** | **ONS LS**  **%** | **E+W**  **%** |
| 20-54 | 142 | 9.3 | 9.4 | 130 | 10.5 | 11.0 | 109 | 3.6 | 3.9 | 1050 | 30.3 | 32.0 |
| 55-64 | 325 | 21.4 | 20.1 | 233 | 18.8 | 17.0 | 624 | 20.5 | 21.0 | 812 | 23.3 | 23.1 |
| 65-74 | 486 | 31.9 | 31.8 | 343 | 27.7 | 25.9 | 1240 | 40.6 | 39.0 | 834 | 24.0 | 21.2 |
| 75+ | 569 | 37.4 | 38.7 | 531 | 43.0 | 46.1 | 1071 | 35.2 | 36.1 | 777 | 22.4 | 23.7 |
| *Total* | *1522* | *100.0* | *100.0* | *1237* | *100.0* | *100.0* | *3044* | *100.0* | *100.0* | *3473* | *100.0* | *100.0* |

**Table S3.** Estimated excess hazard ratios (95% CI) by sex and cancer site across individual-level socio-economic groups as obtained from the model with best fit for Aim 1 (see methods). These excess hazard ratios are also shown in Figure 1. Data source: ONS-LS.

| **SES group** | | **Excess hazard ratio** | **95% CI** |
| --- | --- | --- | --- |
| **Men - colorectal** | | | |
| Education | No qualifications | 1.00 |  |
|  | School-level | 0.892 | 0.698-1.140 |
|  | Apprent/Vocat | 0.737 | 0.563-0.965 |
|  | Degree-level | 0.801 | 0.595-1.077 |
| Occupation | Manual/Tech | 1.00 |  |
|  | Intermediate | 0.989 | 0.784-1.247 |
|  | Manag/Prof | 1.114 | 0.857-1.449 |
| Income | Lowest income | 1.00 |  |
|  | Q2 | 0.792 | 0.611-1.027 |
|  | Q3 | 1.030 | 0.788-1.347 |
|  | Q4 | 0.748 | 0.526-1.064 |
|  | Highest income | 0.771 | 0.500-1.187 |
| **Women - colorectal** | | | |
| Education | No qualifications | 1.00 |  |
|  | School-level | 1.006 | 0.776-1.305 |
|  | Apprent/Vocat | 0.812 | 0.515-1.278 |
|  | Degree-level | 0.840 | 0.591-1.193 |
| Occupation | Manual/Tech | 1.00 |  |
|  | Intermediate | 0.814 | 0.626-1.057 |
|  | Manag/Prof | 0.771 | 0.540-1.101 |
| Income | Lowest income | 1.00 |  |
|  | Q2 | 0.961 | 0.731-1.264 |
|  | Q3 | 0.957 | 0.684-1.339 |
|  | Q4 | 1.173 | 0.781-1.762 |
|  | Highest income | 0.881 | 0.494-1.571 |
| **Men - prostate** | | | |
| Education | No qualifications | 1.00 |  |
|  | School-level | 1.635 | 1.062-2.515 |
|  | Apprent/Vocat | 0.973 | 0.587-1.260 |
|  | Degree-level | 0.857 | 0.481-1.525 |
| Occupation | Manual/Tech | 1.00 |  |
|  | Intermediate | 0.830 | 0.547-1.260 |
|  | Manag/Prof | 0.589 | 0.360-0.965 |
| Income | Lowest income | 1.00 |  |
|  | Q2 | 1.137 | 0.741-1.746 |
|  | Q3 | 1.116 | 0.667-1.865 |
|  | Q4 | 1.213 | 0.626-2.349 |
|  | Highest income | 1.579 | 0.690-3.615 |
| **Women - breast** | | | |
| Education | No qualifications | 1.00 |  |
|  | School-level | 0.852 | 0.618-1.175 |
|  | Apprent/Vocat | 0.770 | 0.455-1.301 |
|  | Degree-level | 0.969 | 0.663-1.415 |
| Occupation | Manual/Tech | 1.00 |  |
|  | Intermediate | 0.950 | 0.684-1.317 |
|  | Manag/Prof | 1.222 | 0.789-1.892 |
| Income | Lowest income | 1.00 |  |
|  | Q2 | 0.769 | 0.529-1.118 |
|  | Q3 | 0.706 | 0.462-1.078 |
|  | Q4 | 0.769 | 0.479-1.235 |
|  | Highest income | 0.522 | 0.286-0.952 |

**Table S4.** Test statistic and associated P-value for likelihood ratio tests of individual socio-economic variables, based on comparison of the best model for each cancer site and sex from Aim 1 with and without the inclusion of each variable. Data source: ONS-LS.

| **Term** | **Likelihood ratio test statistic** | **P-value** |
| --- | --- | --- |
| **Men - colorectal** | | |
| Education | 6.25 | 0.100 |
| Occupation | 0.83 | 0.660 |
| Income | 1.19 | 0.880 |
| **Women - colorectal** | | |
| Education | 1.88 | 0.598 |
| Occupation | 4.70 | 0.095 |
| Income | 2.23 | 0.694 |
| **Men - prostate** | | |
| Education | 8.93 | 0.030 |
| Occupation | 4.64 | 0.098 |
| Income | 7.79 | 0.099 |
| **Women - breast** | | |
| Education | 1.82 | 0.611 |
| Occupation | 1.71 | 0.425 |
| Income | 8.89 | 0.064 |

**Table S5.** Estimated excess hazard ratios (95% CI) by sex and cancer site across individual- and area-level socio-economic groups as obtained from the model with best fit for Aim 2 (see methods). These excess hazard ratios are also shown in Figure 2. Note: excess hazard ratios across occupation groups for prostate and breast cancers are shown within the least and most deprived deprivation ventiles. Data source: ONS-LS.

| **SES group** | | **Excess hazard ratio** | **95% CI** |
| --- | --- | --- | --- |
| **Men - colorectal** | | | |
| Education | No qualifications | 1.00 |  |
|  | School-level | 0.907 | 0.709-1.161 |
|  | Apprent/Vocat | 0.743 | 0.568-0.973 |
|  | Degree-level | 0.821 | 0.609-1.108 |
| Occupation | Manual/Tech | 1.00 |  |
|  | Intermediate | 1.011 | 0.799-1.279 |
|  | Manag/Prof | 1.148 | 0.878-1.501 |
| Income | Lowest income | 1.00 |  |
|  | Q2 | 0.793 | 0.611-1.028 |
|  | Q3 | 1.037 | 0.794-1.356 |
|  | Q4 | 0.756 | 0.532-1.075 |
|  | Highest income | 0.770 | 0.500-1.186 |
| Area | Per ventile | 0.813 | 0.604-1.094 |
| **Women - colorectal** | | | |
| Education | No qualifications | 1.00 |  |
|  | School-level | 1.012 | 0.779-1.313 |
|  | Apprent/Vocat | 0.812 | 0.516-1.279 |
|  | Degree-level | 0.847 | 0.595-1.204 |
| Occupation | Manual/Tech | 1.00 |  |
|  | Intermediate | 0.823 | 0.631-1.073 |
|  | Manag/Prof | 0.781 | 0.545-1.120 |
| Income | Lowest income | 1.00 |  |
|  | Q2 | 0.961 | 0.731-1.263 |
|  | Q3 | 0.957 | 0.684-1.340 |
|  | Q4 | 1.162 | 0.773-1.747 |
|  | Highest income | 0.877 | 0.492-1.564 |
| Area | Per ventile | 0.843 | 0.593-1.197 |
| **Men - prostate** | | | |
| Education | No qualifications | 1.00 |  |
|  | School-level | 1.825 | 1.179-2.823 |
|  | Apprent/Vocat | 1.101 | 0.674-1.800 |
|  | Degree-level | 0.977 | 0.547-1.746 |
| Occupation - most deprived areas | Manual/Tech | 1.00 |  |
|  | Intermediate | 0.974 | 0.467-2.029 |
|  | Manag/Prof | 0.502 | 0.215-1.170 |
| Occupation - least deprived areas | Manual/Tech | 1.00 |  |
|  | Intermediate | 0.986 | 0.397-2.449 |
|  | Manag/Prof | 0.884 | 0.374-2.088 |
| Income | Lowest income | 1.00 |  |
|  | Q2 | 1.199 | 0.785-1.833 |
|  | Q3 | 1.176 | 0.705-1.960 |
|  | Q4 | 1.220 | 0.634-2.349 |
|  | Highest income | 1.676 | 0.758-3.708 |
| Area | Per ventile | 0.918 | 0.515-1.635 |
| **Women - breast** | | | |
| Education | No qualifications | 1.00 |  |
|  | School-level | 0.901 | 0.654-1.241 |
|  | Apprent/Vocat | 0.807 | 0.478-1.363 |
|  | Degree-level | 1.046 | 0.717-1.526 |
| Occupation - most deprived areas | Manual/Tech | 1.00 |  |
|  | Intermediate | 0.581 | 0.312-1.082 |
|  | Manag/Prof | 1.179 | 0.643-2.162 |
| Occupation - least deprived areas | Manual/Tech | 1.00 |  |
|  | Intermediate | 1.557 | 0.874-2.774 |
|  | Manag/Prof | 1.376 | 0.700-2.705 |
| Income | Lowest income | 1.00 |  |
|  | Q2 | 0.789 | 0.545-1.142 |
|  | Q3 | 0.728 | 0.480-1.104 |
|  | Q4 | 0.807 | 0.505-1.287 |
|  | Highest income | 0.572 | 0.315-1.040 |
| Area | Per ventile | 1.00 | 0.686-1.458 |

**Table S6.** Estimates of net survival (95% CI) from the best fit model from Aim 2 for each combination of sex/cancer site. Estimates are shown for each combination of individual-level income, education and occupation socio-economic group, and within the most and least deprived area-level deprivation contexts, and for survival estimated at 1-year and 5-years since diagnosis. Estimates assume a median age of 70 years old. Data source: ONS-LS.

| **Dataset** | **SES group** | | **Most deprived areas** | | | | | | | | **Least deprived areas** | | | | | | | |
| --- | --- | --- | --- | --- | --- | --- | --- | --- | --- | --- | --- | --- | --- | --- | --- | --- | --- | --- |
|  |  |  | **No quals** | | **School** | | **Apprent** | | **Degree** | | **No quals** | | **School** | | **Apprent** | | **Degree** | |
| **1-year net survival** | | | | | | | | | | | | | | | | | | |
| **Men - colorectal** | **Manual** | **Low income** | 0.78 | (0.72-0.82) | 0.8 | (0.73-0.85) | 0.83 | (0.77-0.88) | 0.81 | (0.74-0.87) | 0.81 | (0.76-0.86) | 0.83 | (0.77-0.88) | 0.86 | (0.8-0.9) | 0.84 | (0.78-0.89) |
|  |  | **Q2** | 0.82 | (0.77-0.86) | 0.84 | (0.78-0.88) | 0.86 | (0.81-0.9) | 0.85 | (0.79-0.9) | 0.85 | (0.8-0.89) | 0.86 | (0.81-0.9) | 0.89 | (0.84-0.92) | 0.87 | (0.82-0.91) |
|  |  | **Q3** | 0.77 | (0.71-0.82) | 0.79 | (0.72-0.85) | 0.82 | (0.76-0.87) | 0.81 | (0.73-0.87) | 0.81 | (0.74-0.86) | 0.82 | (0.76-0.87) | 0.85 | (0.8-0.9) | 0.84 | (0.77-0.89) |
|  |  | **Q4** | 0.83 | (0.76-0.88) | 0.84 | (0.77-0.89) | 0.87 | (0.81-0.91) | 0.86 | (0.79-0.9) | 0.86 | (0.79-0.9) | 0.87 | (0.81-0.91) | 0.89 | (0.84-0.93) | 0.88 | (0.82-0.92) |
|  |  | **High income** | 0.82 | (0.74-0.88) | 0.84 | (0.76-0.9) | 0.87 | (0.79-0.92) | 0.85 | (0.78-0.91) | 0.85 | (0.78-0.91) | 0.87 | (0.79-0.92) | 0.89 | (0.82-0.93) | 0.88 | (0.81-0.92) |
|  | **Intermed** | **Low income** | 0.78 | (0.71-0.83) | 0.79 | (0.72-0.85) | 0.83 | (0.76-0.88) | 0.81 | (0.73-0.87) | 0.81 | (0.75-0.86) | 0.83 | (0.77-0.87) | 0.86 | (0.8-0.9) | 0.84 | (0.78-0.89) |
|  |  | **Q2** | 0.82 | (0.76-0.87) | 0.83 | (0.77-0.88) | 0.86 | (0.8-0.9) | 0.85 | (0.78-0.9) | 0.85 | (0.79-0.89) | 0.86 | (0.81-0.9) | 0.88 | (0.84-0.92) | 0.87 | (0.82-0.91) |
|  |  | **Q3** | 0.77 | (0.69-0.83) | 0.79 | (0.71-0.85) | 0.82 | (0.75-0.87) | 0.81 | (0.72-0.87) | 0.81 | (0.74-0.86) | 0.82 | (0.76-0.87) | 0.85 | (0.8-0.89) | 0.84 | (0.77-0.89) |
|  |  | **Q4** | 0.83 | (0.75-0.88) | 0.84 | (0.77-0.89) | 0.87 | (0.8-0.91) | 0.85 | (0.78-0.9) | 0.85 | (0.79-0.9) | 0.87 | (0.81-0.91) | 0.89 | (0.84-0.93) | 0.88 | (0.82-0.92) |
|  |  | **High income** | 0.82 | (0.73-0.88) | 0.84 | (0.75-0.9) | 0.87 | (0.79-0.92) | 0.85 | (0.77-0.91) | 0.85 | (0.77-0.91) | 0.86 | (0.79-0.91) | 0.89 | (0.82-0.93) | 0.88 | (0.81-0.92) |
|  | **Manag** | **Low income** | 0.75 | (0.66-0.82) | 0.77 | (0.68-0.84) | 0.81 | (0.72-0.87) | 0.79 | (0.7-0.86) | 0.79 | (0.71-0.85) | 0.81 | (0.73-0.86) | 0.84 | (0.77-0.89) | 0.82 | (0.75-0.88) |
|  |  | **Q2** | 0.8 | (0.72-0.85) | 0.81 | (0.74-0.87) | 0.84 | (0.78-0.89) | 0.83 | (0.76-0.88) | 0.83 | (0.76-0.88) | 0.84 | (0.79-0.89) | 0.87 | (0.81-0.91) | 0.86 | (0.8-0.9) |
|  |  | **Q3** | 0.74 | (0.66-0.81) | 0.76 | (0.68-0.83) | 0.8 | (0.73-0.86) | 0.78 | (0.7-0.84) | 0.78 | (0.71-0.84) | 0.8 | (0.74-0.85) | 0.83 | (0.77-0.88) | 0.82 | (0.76-0.86) |
|  |  | **Q4** | 0.8 | (0.72-0.86) | 0.82 | (0.75-0.88) | 0.85 | (0.78-0.9) | 0.84 | (0.77-0.89) | 0.84 | (0.77-0.89) | 0.85 | (0.79-0.89) | 0.88 | (0.82-0.91) | 0.86 | (0.82-0.9) |
|  |  | **High income** | 0.8 | (0.71-0.86) | 0.82 | (0.74-0.88) | 0.85 | (0.77-0.9) | 0.83 | (0.76-0.88) | 0.83 | (0.76-0.89) | 0.85 | (0.78-0.9) | 0.87 | (0.81-0.92) | 0.86 | (0.81-0.9) |
| **Women - colorectal** | **Manual** | **Low income** | 0.77 | (0.71-0.82) | 0.76 | (0.68-0.83) | 0.81 | (0.7-0.88) | 0.8 | (0.71-0.86) | 0.78 | (0.72-0.83) | 0.78 | (0.71-0.84) | 0.82 | (0.72-0.89) | 0.81 | (0.73-0.87) |
|  |  | **Q2** | 0.77 | (0.71-0.83) | 0.77 | (0.69-0.83) | 0.81 | (0.71-0.88) | 0.81 | (0.71-0.87) | 0.79 | (0.72-0.84) | 0.79 | (0.71-0.85) | 0.83 | (0.72-0.89) | 0.82 | (0.74-0.88) |
|  |  | **Q3** | 0.78 | (0.7-0.83) | 0.77 | (0.69-0.84) | 0.81 | (0.7-0.89) | 0.81 | (0.71-0.87) | 0.79 | (0.71-0.85) | 0.79 | (0.71-0.85) | 0.83 | (0.72-0.9) | 0.82 | (0.73-0.88) |
|  |  | **Q4** | 0.73 | (0.63-0.81) | 0.73 | (0.62-0.82) | 0.78 | (0.64-0.87) | 0.77 | (0.66-0.85) | 0.75 | (0.64-0.84) | 0.75 | (0.63-0.83) | 0.79 | (0.66-0.88) | 0.79 | (0.68-0.86) |
|  |  | **High income** | 0.79 | (0.66-0.88) | 0.79 | (0.65-0.88) | 0.83 | (0.68-0.91) | 0.82 | (0.7-0.9) | 0.81 | (0.68-0.89) | 0.81 | (0.67-0.89) | 0.84 | (0.7-0.92) | 0.83 | (0.72-0.9) |
|  | **Intermed** | **Low income** | 0.8 | (0.73-0.86) | 0.8 | (0.72-0.86) | 0.84 | (0.74-0.9) | 0.83 | (0.74-0.89) | 0.82 | (0.75-0.87) | 0.82 | (0.74-0.87) | 0.85 | (0.76-0.91) | 0.84 | (0.77-0.9) |
|  |  | **Q2** | 0.81 | (0.75-0.86) | 0.81 | (0.74-0.86) | 0.84 | (0.75-0.9) | 0.84 | (0.76-0.89) | 0.82 | (0.77-0.87) | 0.82 | (0.76-0.87) | 0.86 | (0.77-0.91) | 0.85 | (0.78-0.9) |
|  |  | **Q3** | 0.81 | (0.74-0.87) | 0.81 | (0.73-0.87) | 0.84 | (0.75-0.91) | 0.84 | (0.75-0.9) | 0.83 | (0.76-0.88) | 0.82 | (0.76-0.87) | 0.86 | (0.77-0.91) | 0.85 | (0.78-0.9) |
|  |  | **Q4** | 0.78 | (0.68-0.85) | 0.77 | (0.68-0.84) | 0.81 | (0.7-0.89) | 0.81 | (0.71-0.87) | 0.79 | (0.7-0.86) | 0.79 | (0.7-0.86) | 0.83 | (0.72-0.9) | 0.82 | (0.74-0.88) |
|  |  | **High income** | 0.83 | (0.71-0.9) | 0.82 | (0.7-0.9) | 0.86 | (0.73-0.93) | 0.85 | (0.75-0.91) | 0.84 | (0.73-0.91) | 0.84 | (0.73-0.91) | 0.87 | (0.75-0.93) | 0.86 | (0.77-0.92) |
|  | **Manag** | **Low income** | 0.81 | (0.72-0.88) | 0.81 | (0.71-0.88) | 0.84 | (0.74-0.91) | 0.84 | (0.74-0.9) | 0.83 | (0.74-0.89) | 0.82 | (0.74-0.88) | 0.86 | (0.76-0.92) | 0.85 | (0.77-0.91) |
|  |  | **Q2** | 0.82 | (0.73-0.88) | 0.82 | (0.73-0.88) | 0.85 | (0.75-0.91) | 0.84 | (0.76-0.9) | 0.83 | (0.76-0.89) | 0.83 | (0.76-0.88) | 0.86 | (0.77-0.92) | 0.86 | (0.78-0.91) |
|  |  | **Q3** | 0.82 | (0.73-0.88) | 0.82 | (0.73-0.88) | 0.85 | (0.75-0.91) | 0.85 | (0.76-0.9) | 0.83 | (0.76-0.89) | 0.83 | (0.76-0.88) | 0.86 | (0.77-0.92) | 0.86 | (0.79-0.91) |
|  |  | **Q4** | 0.79 | (0.7-0.85) | 0.78 | (0.7-0.85) | 0.82 | (0.71-0.89) | 0.82 | (0.74-0.87) | 0.8 | (0.72-0.86) | 0.8 | (0.72-0.86) | 0.84 | (0.73-0.9) | 0.83 | (0.76-0.88) |
|  |  | **High income** | 0.83 | (0.74-0.9) | 0.83 | (0.73-0.9) | 0.86 | (0.75-0.93) | 0.86 | (0.78-0.91) | 0.85 | (0.76-0.9) | 0.84 | (0.76-0.9) | 0.87 | (0.77-0.93) | 0.87 | (0.8-0.91) |
| **Men - prostate** | **Manual** | **Low income** | 0.96 | (0.93-0.98) | 0.93 | (0.88-0.96) | 0.96 | (0.92-0.98) | 0.96 | (0.92-0.98) | 0.99 | (0.97-0.99) | 0.98 | (0.95-0.99) | 0.99 | (0.97-0.99) | 0.99 | (0.97-1) |
|  |  | **Q2** | 0.95 | (0.92-0.97) | 0.92 | (0.85-0.96) | 0.95 | (0.91-0.97) | 0.96 | (0.91-0.98) | 0.99 | (0.97-0.99) | 0.97 | (0.95-0.99) | 0.98 | (0.97-0.99) | 0.99 | (0.97-0.99) |
|  |  | **Q3** | 0.96 | (0.92-0.98) | 0.92 | (0.85-0.96) | 0.95 | (0.91-0.97) | 0.96 | (0.91-0.98) | 0.99 | (0.97-0.99) | 0.98 | (0.95-0.99) | 0.99 | (0.97-0.99) | 0.99 | (0.97-0.99) |
|  |  | **Q4** | 0.95 | (0.91-0.98) | 0.92 | (0.84-0.96) | 0.95 | (0.89-0.98) | 0.95 | (0.9-0.98) | 0.99 | (0.97-0.99) | 0.97 | (0.94-0.99) | 0.98 | (0.96-0.99) | 0.99 | (0.97-0.99) |
|  |  | **High income** | 0.94 | (0.86-0.97) | 0.89 | (0.76-0.95) | 0.93 | (0.84-0.97) | 0.94 | (0.86-0.97) | 0.98 | (0.95-0.99) | 0.96 | (0.91-0.99) | 0.98 | (0.95-0.99) | 0.98 | (0.95-0.99) |
|  | **Intermed** | **Low income** | 0.96 | (0.92-0.98) | 0.93 | (0.86-0.97) | 0.96 | (0.91-0.98) | 0.96 | (0.92-0.98) | 0.99 | (0.97-1) | 0.98 | (0.96-0.99) | 0.99 | (0.97-1) | 0.99 | (0.97-1) |
|  |  | **Q2** | 0.96 | (0.91-0.98) | 0.92 | (0.83-0.96) | 0.95 | (0.89-0.98) | 0.96 | (0.9-0.98) | 0.99 | (0.97-0.99) | 0.98 | (0.95-0.99) | 0.99 | (0.96-0.99) | 0.99 | (0.97-0.99) |
|  |  | **Q3** | 0.96 | (0.9-0.98) | 0.92 | (0.83-0.96) | 0.95 | (0.89-0.98) | 0.96 | (0.9-0.98) | 0.99 | (0.97-0.99) | 0.98 | (0.95-0.99) | 0.99 | (0.97-0.99) | 0.99 | (0.97-0.99) |
|  |  | **Q4** | 0.95 | (0.89-0.98) | 0.92 | (0.82-0.97) | 0.95 | (0.88-0.98) | 0.96 | (0.89-0.98) | 0.99 | (0.97-0.99) | 0.97 | (0.94-0.99) | 0.98 | (0.96-0.99) | 0.99 | (0.97-0.99) |
|  |  | **High income** | 0.94 | (0.85-0.98) | 0.89 | (0.73-0.96) | 0.93 | (0.82-0.97) | 0.94 | (0.84-0.98) | 0.98 | (0.95-0.99) | 0.97 | (0.91-0.99) | 0.98 | (0.94-0.99) | 0.98 | (0.95-0.99) |
|  | **Manag** | **Low income** | 0.98 | (0.95-0.99) | 0.97 | (0.92-0.99) | 0.98 | (0.94-0.99) | 0.98 | (0.95-0.99) | 0.99 | (0.98-1) | 0.98 | (0.96-0.99) | 0.99 | (0.97-1) | 0.99 | (0.98-1) |
|  |  | **Q2** | 0.98 | (0.94-0.99) | 0.96 | (0.9-0.98) | 0.97 | (0.94-0.99) | 0.98 | (0.94-0.99) | 0.99 | (0.97-0.99) | 0.98 | (0.95-0.99) | 0.99 | (0.97-0.99) | 0.99 | (0.97-0.99) |
|  |  | **Q3** | 0.98 | (0.94-0.99) | 0.96 | (0.91-0.98) | 0.98 | (0.94-0.99) | 0.98 | (0.95-0.99) | 0.99 | (0.97-0.99) | 0.98 | (0.96-0.99) | 0.99 | (0.97-0.99) | 0.99 | (0.98-0.99) |
|  |  | **Q4** | 0.98 | (0.94-0.99) | 0.96 | (0.9-0.98) | 0.97 | (0.93-0.99) | 0.98 | (0.94-0.99) | 0.99 | (0.97-0.99) | 0.98 | (0.95-0.99) | 0.99 | (0.97-0.99) | 0.99 | (0.97-0.99) |
|  |  | **High income** | 0.97 | (0.91-0.99) | 0.94 | (0.86-0.98) | 0.96 | (0.91-0.99) | 0.97 | (0.92-0.99) | 0.98 | (0.96-0.99) | 0.97 | (0.93-0.99) | 0.98 | (0.96-0.99) | 0.98 | (0.96-0.99) |
| **Women - breast** | **Manual** | **Low income** | 0.93 | (0.9-0.95) | 0.94 | (0.9-0.96) | 0.94 | (0.9-0.97) | 0.93 | (0.88-0.96) | 0.97 | (0.95-0.98) | 0.97 | (0.95-0.98) | 0.98 | (0.95-0.99) | 0.97 | (0.94-0.98) |
|  |  | **Q2** | 0.94 | (0.92-0.96) | 0.95 | (0.92-0.97) | 0.96 | (0.92-0.98) | 0.94 | (0.9-0.97) | 0.98 | (0.96-0.99) | 0.98 | (0.96-0.99) | 0.98 | (0.96-0.99) | 0.98 | (0.96-0.99) |
|  |  | **Q3** | 0.95 | (0.92-0.97) | 0.95 | (0.92-0.97) | 0.96 | (0.92-0.98) | 0.95 | (0.91-0.97) | 0.98 | (0.96-0.99) | 0.98 | (0.97-0.99) | 0.98 | (0.96-0.99) | 0.98 | (0.96-0.99) |
|  |  | **Q4** | 0.94 | (0.91-0.97) | 0.95 | (0.91-0.97) | 0.95 | (0.91-0.98) | 0.94 | (0.9-0.97) | 0.98 | (0.96-0.99) | 0.98 | (0.96-0.99) | 0.98 | (0.96-0.99) | 0.97 | (0.95-0.99) |
|  |  | **High income** | 0.96 | (0.92-0.98) | 0.96 | (0.93-0.98) | 0.97 | (0.93-0.99) | 0.96 | (0.92-0.98) | 0.98 | (0.96-0.99) | 0.98 | (0.97-0.99) | 0.99 | (0.97-0.99) | 0.98 | (0.96-0.99) |
|  | **Intermed** | **Low income** | 0.96 | (0.92-0.98) | 0.96 | (0.93-0.98) | 0.97 | (0.93-0.98) | 0.96 | (0.92-0.98) | 0.95 | (0.92-0.97) | 0.96 | (0.93-0.97) | 0.96 | (0.93-0.98) | 0.95 | (0.92-0.97) |
|  |  | **Q2** | 0.97 | (0.94-0.98) | 0.97 | (0.95-0.98) | 0.97 | (0.95-0.99) | 0.97 | (0.94-0.98) | 0.96 | (0.94-0.98) | 0.97 | (0.95-0.98) | 0.97 | (0.94-0.98) | 0.96 | (0.94-0.98) |
|  |  | **Q3** | 0.97 | (0.94-0.98) | 0.97 | (0.95-0.99) | 0.98 | (0.95-0.99) | 0.97 | (0.94-0.98) | 0.97 | (0.94-0.98) | 0.97 | (0.95-0.98) | 0.97 | (0.95-0.99) | 0.96 | (0.94-0.98) |
|  |  | **Q4** | 0.97 | (0.94-0.98) | 0.97 | (0.94-0.98) | 0.97 | (0.94-0.99) | 0.97 | (0.93-0.98) | 0.96 | (0.94-0.98) | 0.97 | (0.94-0.98) | 0.97 | (0.94-0.98) | 0.96 | (0.93-0.98) |
|  |  | **High income** | 0.98 | (0.95-0.99) | 0.98 | (0.96-0.99) | 0.98 | (0.96-0.99) | 0.98 | (0.95-0.99) | 0.97 | (0.95-0.99) | 0.98 | (0.95-0.99) | 0.98 | (0.95-0.99) | 0.97 | (0.95-0.98) |
|  | **Manag** | **Low income** | 0.92 | (0.85-0.95) | 0.93 | (0.87-0.96) | 0.93 | (0.86-0.97) | 0.91 | (0.84-0.95) | 0.96 | (0.92-0.98) | 0.96 | (0.93-0.98) | 0.97 | (0.93-0.98) | 0.96 | (0.92-0.98) |
|  |  | **Q2** | 0.93 | (0.89-0.96) | 0.94 | (0.9-0.97) | 0.95 | (0.89-0.97) | 0.93 | (0.88-0.96) | 0.97 | (0.94-0.98) | 0.97 | (0.95-0.98) | 0.97 | (0.95-0.99) | 0.97 | (0.94-0.98) |
|  |  | **Q3** | 0.94 | (0.89-0.97) | 0.95 | (0.9-0.97) | 0.95 | (0.9-0.98) | 0.94 | (0.89-0.97) | 0.97 | (0.94-0.98) | 0.97 | (0.95-0.98) | 0.98 | (0.95-0.99) | 0.97 | (0.94-0.98) |
|  |  | **Q4** | 0.93 | (0.89-0.96) | 0.94 | (0.9-0.96) | 0.95 | (0.89-0.97) | 0.93 | (0.88-0.96) | 0.97 | (0.94-0.98) | 0.97 | (0.95-0.98) | 0.97 | (0.95-0.99) | 0.97 | (0.94-0.98) |
|  |  | **High income** | 0.95 | (0.91-0.97) | 0.96 | (0.92-0.98) | 0.96 | (0.92-0.98) | 0.95 | (0.91-0.97) | 0.98 | (0.96-0.99) | 0.98 | (0.96-0.99) | 0.98 | (0.96-0.99) | 0.98 | (0.96-0.98) |
| **5-year net survival** | | | | | | | | | | | | | | | | | | |
| **Men - colorectal** | **Manual** | **Low income** | 0.54 | (0.45-0.62) | 0.57 | (0.46-0.66) | 0.63 | (0.53-0.72) | 0.6 | (0.48-0.71) | 0.6 | (0.5-0.69) | 0.63 | (0.53-0.72) | 0.69 | (0.58-0.77) | 0.66 | (0.54-0.75) |
|  |  | **Q2** | 0.61 | (0.53-0.69) | 0.64 | (0.54-0.73) | 0.7 | (0.6-0.77) | 0.67 | (0.56-0.76) | 0.67 | (0.58-0.75) | 0.69 | (0.6-0.77) | 0.74 | (0.65-0.81) | 0.72 | (0.62-0.8) |
|  |  | **Q3** | 0.53 | (0.43-0.62) | 0.56 | (0.45-0.66) | 0.62 | (0.51-0.71) | 0.59 | (0.46-0.7) | 0.59 | (0.49-0.68) | 0.62 | (0.51-0.71) | 0.68 | (0.57-0.76) | 0.65 | (0.53-0.74) |
|  |  | **Q4** | 0.63 | (0.51-0.72) | 0.65 | (0.53-0.75) | 0.71 | (0.6-0.79) | 0.68 | (0.56-0.78) | 0.68 | (0.57-0.77) | 0.71 | (0.6-0.79) | 0.75 | (0.65-0.83) | 0.73 | (0.62-0.81) |
|  |  | **High income** | 0.62 | (0.48-0.73) | 0.65 | (0.5-0.76) | 0.7 | (0.57-0.8) | 0.68 | (0.54-0.78) | 0.68 | (0.54-0.78) | 0.7 | (0.56-0.8) | 0.75 | (0.62-0.84) | 0.73 | (0.6-0.82) |
|  | **Intermed** | **Low income** | 0.54 | (0.43-0.63) | 0.57 | (0.45-0.67) | 0.63 | (0.51-0.73) | 0.6 | (0.46-0.71) | 0.6 | (0.5-0.69) | 0.63 | (0.52-0.72) | 0.68 | (0.58-0.77) | 0.66 | (0.54-0.75) |
|  |  | **Q2** | 0.61 | (0.5-0.7) | 0.64 | (0.52-0.73) | 0.69 | (0.59-0.78) | 0.67 | (0.54-0.76) | 0.67 | (0.57-0.75) | 0.69 | (0.59-0.77) | 0.74 | (0.65-0.81) | 0.72 | (0.61-0.8) |
|  |  | **Q3** | 0.52 | (0.41-0.63) | 0.56 | (0.43-0.66) | 0.62 | (0.5-0.72) | 0.59 | (0.45-0.7) | 0.59 | (0.48-0.68) | 0.62 | (0.51-0.71) | 0.67 | (0.57-0.76) | 0.65 | (0.53-0.74) |
|  |  | **Q4** | 0.62 | (0.49-0.73) | 0.65 | (0.52-0.75) | 0.7 | (0.58-0.8) | 0.68 | (0.55-0.78) | 0.68 | (0.56-0.77) | 0.7 | (0.6-0.79) | 0.75 | (0.65-0.83) | 0.73 | (0.62-0.81) |
|  |  | **High income** | 0.62 | (0.47-0.74) | 0.65 | (0.49-0.76) | 0.7 | (0.56-0.8) | 0.67 | (0.53-0.78) | 0.67 | (0.53-0.78) | 0.7 | (0.56-0.8) | 0.75 | (0.62-0.84) | 0.72 | (0.6-0.81) |
|  | **Manag** | **Low income** | 0.49 | (0.36-0.61) | 0.53 | (0.39-0.65) | 0.59 | (0.45-0.71) | 0.56 | (0.42-0.68) | 0.56 | (0.43-0.67) | 0.59 | (0.47-0.69) | 0.65 | (0.52-0.75) | 0.62 | (0.5-0.72) |
|  |  | **Q2** | 0.57 | (0.45-0.68) | 0.6 | (0.48-0.7) | 0.66 | (0.54-0.76) | 0.63 | (0.51-0.73) | 0.63 | (0.52-0.72) | 0.66 | (0.55-0.74) | 0.71 | (0.61-0.79) | 0.68 | (0.58-0.77) |
|  |  | **Q3** | 0.48 | (0.36-0.59) | 0.51 | (0.39-0.62) | 0.58 | (0.46-0.68) | 0.55 | (0.42-0.66) | 0.55 | (0.44-0.64) | 0.58 | (0.48-0.67) | 0.64 | (0.53-0.73) | 0.61 | (0.51-0.69) |
|  |  | **Q4** | 0.58 | (0.45-0.7) | 0.61 | (0.49-0.72) | 0.67 | (0.55-0.77) | 0.64 | (0.52-0.74) | 0.64 | (0.53-0.74) | 0.67 | (0.57-0.75) | 0.72 | (0.62-0.8) | 0.7 | (0.61-0.77) |
|  |  | **High income** | 0.58 | (0.44-0.7) | 0.61 | (0.47-0.72) | 0.67 | (0.53-0.77) | 0.64 | (0.52-0.74) | 0.64 | (0.51-0.74) | 0.67 | (0.55-0.76) | 0.72 | (0.6-0.8) | 0.69 | (0.59-0.77) |
| **Women - colorectal** | **Manual** | **Low income** | 0.57 | (0.48-0.65) | 0.56 | (0.45-0.66) | 0.63 | (0.47-0.75) | 0.62 | (0.48-0.73) | 0.59 | (0.5-0.68) | 0.59 | (0.48-0.69) | 0.66 | (0.5-0.77) | 0.64 | (0.51-0.75) |
|  |  | **Q2** | 0.58 | (0.48-0.66) | 0.58 | (0.46-0.68) | 0.64 | (0.48-0.77) | 0.63 | (0.49-0.74) | 0.61 | (0.51-0.69) | 0.6 | (0.49-0.7) | 0.67 | (0.51-0.79) | 0.66 | (0.52-0.76) |
|  |  | **Q3** | 0.58 | (0.47-0.68) | 0.58 | (0.45-0.69) | 0.64 | (0.47-0.77) | 0.63 | (0.48-0.75) | 0.61 | (0.49-0.71) | 0.6 | (0.48-0.71) | 0.67 | (0.5-0.79) | 0.66 | (0.51-0.77) |
|  |  | **Q4** | 0.52 | (0.37-0.64) | 0.51 | (0.36-0.65) | 0.59 | (0.39-0.74) | 0.57 | (0.41-0.7) | 0.55 | (0.39-0.68) | 0.54 | (0.38-0.68) | 0.61 | (0.41-0.76) | 0.6 | (0.44-0.73) |
|  |  | **High income** | 0.61 | (0.42-0.75) | 0.6 | (0.4-0.76) | 0.67 | (0.44-0.82) | 0.66 | (0.47-0.79) | 0.63 | (0.44-0.78) | 0.63 | (0.43-0.78) | 0.69 | (0.47-0.84) | 0.68 | (0.5-0.81) |
|  | **Intermed** | **Low income** | 0.63 | (0.51-0.73) | 0.62 | (0.49-0.73) | 0.68 | (0.52-0.8) | 0.67 | (0.52-0.79) | 0.65 | (0.54-0.74) | 0.65 | (0.53-0.74) | 0.71 | (0.56-0.81) | 0.7 | (0.57-0.79) |
|  |  | **Q2** | 0.64 | (0.54-0.73) | 0.64 | (0.52-0.73) | 0.7 | (0.55-0.8) | 0.68 | (0.55-0.79) | 0.66 | (0.57-0.74) | 0.66 | (0.57-0.74) | 0.72 | (0.58-0.82) | 0.71 | (0.59-0.79) |
|  |  | **Q3** | 0.64 | (0.52-0.74) | 0.64 | (0.51-0.74) | 0.7 | (0.54-0.81) | 0.69 | (0.54-0.79) | 0.66 | (0.56-0.75) | 0.66 | (0.56-0.75) | 0.72 | (0.57-0.82) | 0.71 | (0.58-0.8) |
|  |  | **Q4** | 0.58 | (0.44-0.7) | 0.58 | (0.44-0.7) | 0.64 | (0.46-0.78) | 0.63 | (0.49-0.75) | 0.61 | (0.47-0.72) | 0.6 | (0.47-0.72) | 0.67 | (0.49-0.79) | 0.66 | (0.52-0.76) |
|  |  | **High income** | 0.66 | (0.48-0.79) | 0.66 | (0.47-0.8) | 0.72 | (0.51-0.85) | 0.71 | (0.54-0.82) | 0.69 | (0.51-0.81) | 0.68 | (0.51-0.81) | 0.74 | (0.54-0.86) | 0.73 | (0.58-0.83) |
|  | **Manag** | **Low income** | 0.64 | (0.49-0.76) | 0.64 | (0.48-0.76) | 0.7 | (0.52-0.82) | 0.69 | (0.53-0.8) | 0.67 | (0.53-0.77) | 0.66 | (0.53-0.77) | 0.72 | (0.56-0.83) | 0.71 | (0.57-0.81) |
|  |  | **Q2** | 0.65 | (0.52-0.76) | 0.65 | (0.51-0.76) | 0.71 | (0.54-0.82) | 0.7 | (0.55-0.8) | 0.68 | (0.55-0.77) | 0.67 | (0.55-0.77) | 0.73 | (0.58-0.83) | 0.72 | (0.59-0.81) |
|  |  | **Q3** | 0.65 | (0.52-0.76) | 0.65 | (0.52-0.76) | 0.71 | (0.54-0.82) | 0.7 | (0.56-0.8) | 0.68 | (0.56-0.77) | 0.67 | (0.56-0.77) | 0.73 | (0.58-0.83) | 0.72 | (0.6-0.81) |
|  |  | **Q4** | 0.6 | (0.46-0.71) | 0.59 | (0.46-0.7) | 0.66 | (0.49-0.78) | 0.65 | (0.53-0.74) | 0.62 | (0.49-0.73) | 0.62 | (0.5-0.72) | 0.68 | (0.52-0.8) | 0.67 | (0.56-0.76) |
|  |  | **High income** | 0.68 | (0.52-0.79) | 0.68 | (0.52-0.79) | 0.73 | (0.55-0.85) | 0.72 | (0.59-0.81) | 0.7 | (0.56-0.81) | 0.7 | (0.56-0.8) | 0.75 | (0.58-0.86) | 0.74 | (0.63-0.82) |
| **Men - prostate** | **Manual** | **Low income** | 0.86 | (0.78-0.91) | 0.76 | (0.62-0.86) | 0.85 | (0.74-0.92) | 0.86 | (0.74-0.93) | 0.96 | (0.91-0.98) | 0.92 | (0.84-0.96) | 0.95 | (0.9-0.98) | 0.96 | (0.9-0.98) |
|  |  | **Q2** | 0.84 | (0.74-0.9) | 0.72 | (0.56-0.83) | 0.82 | (0.69-0.9) | 0.84 | (0.7-0.92) | 0.95 | (0.9-0.97) | 0.91 | (0.82-0.95) | 0.94 | (0.89-0.97) | 0.95 | (0.89-0.98) |
|  |  | **Q3** | 0.84 | (0.74-0.91) | 0.73 | (0.56-0.84) | 0.82 | (0.7-0.9) | 0.84 | (0.71-0.92) | 0.95 | (0.89-0.98) | 0.91 | (0.82-0.96) | 0.94 | (0.88-0.97) | 0.95 | (0.89-0.98) |
|  |  | **Q4** | 0.83 | (0.7-0.91) | 0.72 | (0.52-0.85) | 0.82 | (0.66-0.91) | 0.84 | (0.68-0.92) | 0.95 | (0.88-0.98) | 0.9 | (0.8-0.96) | 0.94 | (0.87-0.97) | 0.95 | (0.88-0.98) |
|  |  | **High income** | 0.78 | (0.58-0.89) | 0.63 | (0.36-0.82) | 0.76 | (0.53-0.89) | 0.78 | (0.56-0.9) | 0.93 | (0.82-0.97) | 0.87 | (0.71-0.95) | 0.92 | (0.81-0.97) | 0.93 | (0.82-0.97) |
|  | **Intermed** | **Low income** | 0.87 | (0.75-0.93) | 0.77 | (0.58-0.88) | 0.85 | (0.7-0.93) | 0.87 | (0.72-0.94) | 0.96 | (0.91-0.98) | 0.92 | (0.84-0.96) | 0.95 | (0.89-0.98) | 0.96 | (0.9-0.98) |
|  |  | **Q2** | 0.84 | (0.7-0.92) | 0.73 | (0.51-0.86) | 0.83 | (0.65-0.92) | 0.84 | (0.67-0.93) | 0.95 | (0.89-0.98) | 0.91 | (0.81-0.96) | 0.94 | (0.87-0.98) | 0.95 | (0.89-0.98) |
|  |  | **Q3** | 0.84 | (0.69-0.92) | 0.73 | (0.51-0.87) | 0.83 | (0.66-0.92) | 0.85 | (0.68-0.93) | 0.95 | (0.89-0.98) | 0.91 | (0.82-0.96) | 0.94 | (0.88-0.98) | 0.95 | (0.89-0.98) |
|  |  | **Q4** | 0.84 | (0.66-0.93) | 0.72 | (0.47-0.87) | 0.82 | (0.62-0.92) | 0.84 | (0.66-0.93) | 0.95 | (0.87-0.98) | 0.91 | (0.79-0.96) | 0.94 | (0.86-0.98) | 0.95 | (0.88-0.98) |
|  |  | **High income** | 0.78 | (0.54-0.91) | 0.64 | (0.32-0.84) | 0.77 | (0.49-0.91) | 0.79 | (0.53-0.92) | 0.93 | (0.82-0.97) | 0.87 | (0.7-0.95) | 0.92 | (0.8-0.97) | 0.93 | (0.82-0.97) |
|  | **Manag** | **Low income** | 0.93 | (0.83-0.97) | 0.87 | (0.73-0.94) | 0.92 | (0.81-0.97) | 0.93 | (0.83-0.97) | 0.96 | (0.92-0.98) | 0.93 | (0.86-0.97) | 0.96 | (0.9-0.98) | 0.96 | (0.92-0.98) |
|  |  | **Q2** | 0.91 | (0.81-0.96) | 0.85 | (0.69-0.93) | 0.91 | (0.78-0.96) | 0.92 | (0.81-0.97) | 0.95 | (0.9-0.98) | 0.92 | (0.84-0.96) | 0.95 | (0.89-0.98) | 0.95 | (0.91-0.98) |
|  |  | **Q3** | 0.92 | (0.81-0.96) | 0.85 | (0.7-0.93) | 0.91 | (0.79-0.96) | 0.92 | (0.82-0.96) | 0.95 | (0.91-0.98) | 0.92 | (0.85-0.96) | 0.95 | (0.9-0.98) | 0.96 | (0.91-0.98) |
|  |  | **Q4** | 0.91 | (0.8-0.96) | 0.85 | (0.68-0.93) | 0.9 | (0.78-0.96) | 0.92 | (0.81-0.96) | 0.95 | (0.9-0.98) | 0.92 | (0.84-0.96) | 0.95 | (0.89-0.98) | 0.95 | (0.91-0.98) |
|  |  | **High income** | 0.88 | (0.72-0.95) | 0.8 | (0.57-0.91) | 0.87 | (0.69-0.95) | 0.89 | (0.73-0.95) | 0.94 | (0.86-0.97) | 0.89 | (0.78-0.94) | 0.93 | (0.84-0.97) | 0.94 | (0.87-0.97) |
| **Women - breast** | **Manual** | **Low income** | 0.77 | (0.69-0.83) | 0.79 | (0.69-0.86) | 0.81 | (0.68-0.89) | 0.76 | (0.63-0.85) | 0.89 | (0.83-0.93) | 0.9 | (0.84-0.94) | 0.91 | (0.84-0.96) | 0.89 | (0.81-0.94) |
|  |  | **Q2** | 0.81 | (0.73-0.87) | 0.83 | (0.74-0.89) | 0.85 | (0.74-0.91) | 0.81 | (0.7-0.88) | 0.92 | (0.87-0.95) | 0.92 | (0.87-0.95) | 0.93 | (0.87-0.96) | 0.91 | (0.85-0.95) |
|  |  | **Q3** | 0.83 | (0.74-0.89) | 0.84 | (0.75-0.9) | 0.86 | (0.75-0.92) | 0.82 | (0.71-0.89) | 0.92 | (0.87-0.95) | 0.93 | (0.88-0.96) | 0.94 | (0.88-0.97) | 0.92 | (0.86-0.95) |
|  |  | **Q4** | 0.81 | (0.7-0.88) | 0.83 | (0.72-0.9) | 0.84 | (0.71-0.92) | 0.8 | (0.67-0.88) | 0.91 | (0.85-0.95) | 0.92 | (0.86-0.96) | 0.93 | (0.86-0.97) | 0.91 | (0.84-0.95) |
|  |  | **High income** | 0.86 | (0.75-0.92) | 0.87 | (0.76-0.93) | 0.89 | (0.77-0.95) | 0.86 | (0.73-0.92) | 0.94 | (0.88-0.97) | 0.94 | (0.89-0.97) | 0.95 | (0.89-0.98) | 0.94 | (0.87-0.97) |
|  | **Intermed** | **Low income** | 0.86 | (0.75-0.92) | 0.87 | (0.77-0.93) | 0.88 | (0.77-0.94) | 0.85 | (0.73-0.92) | 0.84 | (0.75-0.9) | 0.86 | (0.77-0.91) | 0.87 | (0.76-0.93) | 0.83 | (0.73-0.9) |
|  |  | **Q2** | 0.89 | (0.81-0.94) | 0.9 | (0.82-0.94) | 0.91 | (0.82-0.95) | 0.88 | (0.79-0.94) | 0.87 | (0.8-0.92) | 0.88 | (0.82-0.92) | 0.9 | (0.81-0.94) | 0.87 | (0.79-0.92) |
|  |  | **Q3** | 0.9 | (0.82-0.94) | 0.91 | (0.83-0.95) | 0.91 | (0.83-0.96) | 0.89 | (0.8-0.94) | 0.88 | (0.82-0.92) | 0.89 | (0.84-0.93) | 0.9 | (0.83-0.95) | 0.88 | (0.8-0.92) |
|  |  | **Q4** | 0.88 | (0.79-0.94) | 0.9 | (0.81-0.94) | 0.91 | (0.81-0.95) | 0.88 | (0.78-0.94) | 0.87 | (0.79-0.92) | 0.88 | (0.82-0.92) | 0.89 | (0.8-0.94) | 0.86 | (0.79-0.92) |
|  |  | **High income** | 0.92 | (0.84-0.96) | 0.92 | (0.85-0.96) | 0.93 | (0.85-0.97) | 0.91 | (0.83-0.96) | 0.91 | (0.83-0.95) | 0.91 | (0.85-0.95) | 0.92 | (0.84-0.96) | 0.9 | (0.83-0.95) |
|  | **Manag** | **Low income** | 0.73 | (0.57-0.85) | 0.76 | (0.59-0.86) | 0.78 | (0.59-0.89) | 0.72 | (0.54-0.84) | 0.86 | (0.75-0.92) | 0.87 | (0.77-0.93) | 0.88 | (0.77-0.94) | 0.85 | (0.74-0.92) |
|  |  | **Q2** | 0.78 | (0.65-0.87) | 0.8 | (0.67-0.89) | 0.82 | (0.67-0.91) | 0.78 | (0.63-0.87) | 0.89 | (0.8-0.94) | 0.9 | (0.82-0.94) | 0.91 | (0.82-0.95) | 0.88 | (0.8-0.93) |
|  |  | **Q3** | 0.8 | (0.67-0.88) | 0.82 | (0.7-0.89) | 0.83 | (0.69-0.92) | 0.79 | (0.65-0.88) | 0.89 | (0.82-0.94) | 0.9 | (0.84-0.94) | 0.91 | (0.83-0.96) | 0.89 | (0.82-0.94) |
|  |  | **Q4** | 0.78 | (0.65-0.86) | 0.8 | (0.68-0.88) | 0.82 | (0.67-0.9) | 0.77 | (0.64-0.86) | 0.88 | (0.81-0.93) | 0.89 | (0.83-0.93) | 0.91 | (0.83-0.95) | 0.88 | (0.82-0.92) |
|  |  | **High income** | 0.84 | (0.73-0.91) | 0.85 | (0.75-0.92) | 0.87 | (0.74-0.93) | 0.83 | (0.72-0.9) | 0.92 | (0.86-0.95) | 0.92 | (0.87-0.95) | 0.93 | (0.87-0.97) | 0.91 | (0.87-0.94) |
